# The Treg/Th17 Imbalance in Patients with Obstructive Sleep Apnoea Syndrome

**DOI:** 10.1155/2012/815308

**Published:** 2012-12-31

**Authors:** Jin Ye, Hui Liu, Gehua Zhang, Peng Li, Zhiyuan Wang, Shaotong Huang, Qintai Yang, Yuan Li

**Affiliations:** ^1^Sleep Disorders Centre and Department of Otolaryngology—Head and Neck Surgery, The Third Affiliated Hospital, Sun Yat-sen University, 600 Tianhe Street, Guangzhou, Guangdong 510630, China; ^2^Division of Pulmonary and Critical Care, Department of Internal Medicine, The Third Affiliated Hospital, Sun Yat-sen University, 600 Tianhe Street, Guangzhou, Guangdong 510630, China; ^3^Sleep Disorders Centre, The Third Affiliated Hospital, Sun Yat-sen University, 600 Tianhe Street, Guangzhou, Guangdong 510630, China

## Abstract

Obstructive sleep apnoea syndrome (OSAS) is a chronic inflammatory disease regulated by T lymphocytes. Our purpose is to assess the pattern of Th17 cells and CD4^+^CD25^+^Foxp3^+^ regulatory T (Treg) cells in peripheral blood of patients with OSAS. Fourty-four OSAS men and 20 healthy volunteers were enrolled. Twenty-three patients were classified into mild to moderate group and 21 cases were classified into severe group according to the severity of OSAS. We detected the frequencies of Th17 and Treg and related serum cytokines secretion and expressions of key transcription factors. OSAS patients revealed significant increase in peripheral Th17 number, Th17-related cytokines (IL-17 and IL-6), and ROR**γ**t mRNA levels. They also presented a significant decrease in Treg number, Treg-related cytokines (TGF-**β**_1_), and Foxp3 mRNA levels as compared with normal persons. As a result, the Th17/Treg ratios were markedly more upregulated in OSAS patients than those in control group. Furthermore, the Th17/Treg ratio was positively related to the severity of OSAS and serum levels of C-reactive protein. The development of OSAS may be associated with peripheral Th17/Treg imbalance and characterized by a proinflammatory cytokine microenvironment. These results opened an alternative explanation for the substantial activation of immune cells in OSAS and the development of related complications.

## 1. Introduction

Obstructive sleep apnoea syndrome (OSAS) is a highly prevalent disease and is recognized as a major public health burden. OSAS is characterized by repeated events of partial or complete upper airway obstruction during sleep that lead to disruption of normal ventilation, hypoxemia, and sleep fragmentation. Although the basic mechanisms mediating this association are likely multifactorial and remain to be fully elucidated, the cumulative burden of a chronic and low-grade systemic inflammation has emerged as the most likely contributor to the occurrence and magnitude of OSAS associated morbidity [1–4]. Accumulating evidence in the past decade has corroborated the close association between OSAS, inflammation and various cardiovascular morbidities [5, 6].

Sleep is characterized by a specific regulation of the endocrine and autonomic nervous system, and thereby sleep is supposed to exert a systemic control over immune function [7, 8]. OSAS is involving various immune cells, particularly T lymphocytes [9, 10]. Activation of T lymphocytes is among the crucial steps leading to the release of inflammatory mediators and adhesion molecules. T cells play a significant role in atherogenesis and plaque development via cytokine production and by directly contributing to vascular injury [11]. In brief, investigating how this breathing disorder modulates immune responses may facilitate understanding the pathogenesis of OSAS in relation with its complications.

Alberti et al. found a prevailing activation of the Th1-type cytokine pattern in OSAS patients by measuring plasma cytokine levels in OSAS patients [12]. Conversely, Dyugovskaya et al. reported a shift in Th1/Th2 cytokine expression towards Th2 dominance in patients with OSAS [10]. In contrast, Dimitrov et al. revealed that, compared with wakefulness, early nocturnal sleep induced a shift in the Th1/Th2 cytokine balance towards increased Th1 activity, as indicated by an increased ratio of IFN-*γ*/IL-4 producing T helper cells. However, the Th1 shift was only of moderate size and replaced by Th2 dominance during late sleep [13]. To date, only a few paper has explored the Th1/Th2 balance in OSAS patients with incompatible results.

There has recently been a minirevolution in the basic understanding of immunology following the discovery of two new subsets of T helper cells, T regulatory (Treg) and Th17, which have opposite effects on autoimmunity and inflammation. Th17 cells expressing retinoic acid related orphan receptor *γ*t (ROR*γ*t) play critical roles in the development of autoimmunity and allergic reactions by producing IL-17 and IL-6. Th17 cell is a key effector in the immune response and play critical roles in the development of autoimmunity by producing IL-17 and, to a lesser extent, TNF-*α* and IL-6. While Treg cells expressing the forkhead/winged helix transcription factor (Foxp3) orchestrate the overall immune response and play a role in maintaining peripheral immune tolerance by contact-dependent suppression or releasing anti-inflammatory cytokines, such as interleukin (IL)-10 and transforming growth factor (TGF)-*β*_1_ and regulating the activity of the effector T cells. Therefore, the Th17/Treg balance may control the development of autoimmunity and inflammation, and Th17/Treg imbalance is proved to be wide existed in human cancer, inflammatory and autoimmune diseases [14–17]. Therefore, we hypothesize that circulating Treg/Th17 imbalance may also present in OSAS patients, which is related with systemic inflammation and important in the pathogenesis of OSAS.

To the best of our knowledge, a possible role for the Th17/Treg axis in OSAS has never been elucidated in OSAS. Thus, the purpose is to test the hypothesis that the balance of Th17 and Treg frequencies in the peripheral circulation is disturbed in patients with varying degrees of OSAS. We evaluated plasma levels of Th17- and Treg-related cytokines and mRNA expression of relevant transcription factors (ROR*γ*t and Foxp3) in peripheral blood monouclear cells (PBMC). As a sensitive marker of inflammation, C-reactive protein (CRP) is believed to be both a by-product and a mediator of the low-grade inflammation that occurs in OSAS. Therefore, we explored the potential correlation of Th17/Treg imbalance with serum CRP level and the severity of OSAS.

## 2. Materials and Methods

### 2.1. Subjects and Protocol

Consecutive men (age ⩾18 and <70 years) with newly confirmed OSAS by overnight polysomnography (PSG) were initially recruited. All of them had been referred to the Sleep Disorders Center, 3rd affiliated hospital of Sun Yat-sen University between Jan. 2008 to Dec. 2011, with symptoms suggesting sleep related breathing disorders and had never been previously diagnosed or treated for OSAS.

Patients with a history of chronic or recent (⩽1 month) clinically significant infectious or inflammatory condition including asthma, trauma, vaccination, any invasive medical/surgical (⩽3 months), or dental (⩽1 month) procedure were eliminated. Patients with morbid obesity (body mass index (BMI) > 35 kg/m^2^), hypertension, coronary artery disease, heart failure, a history of stroke, diabetes mellitus, chronic obstructive or restrictive pulmonary disease, chronic renal disease, dyslipidemias, or pharmacologically treated depression were ineligible for the study. Current smokers and exsmokers who smoked within 12 months before the start of current study were excluded. Patients receiving medications or nutritional supplements and night shift workers also were ineligible. Patients with sleep disorders such as upper airway resistance syndrome, central sleep apnea syndrome, periodic limbs movement, or narcolepsy were also removed from final analysis.

We recruited control subjects from the community at the same time. Control subjects were nonsmoking healthy subjects who had an AHI lower than 5 and no complaint of sleep apnea. They have no chronic diseases mentioned above. Control subjects were matched to OSAS patients for gender (only male), age (within 4 years), and BMI (within 15%). Except for possible obesity, all control subjects had a normal physical examination and laboratory tests. All control subjects underwent PSG to exclude the presence of sleep-disordered breathing.

All subjects completed a standardized questionnaire including the Epworth sleepiness scale (ESS) score [18], demographic data, and clinical history. The study was approved by the institutional review board of the Institutional Review Board of Sun Yat-sen University. All patients gave their written informed consent.

### 2.2. Data Collection

At baseline, medical history was recorded and a physical examination was performed. Anthropometric data (age, BMI, neck circumferences) along with daytime habits, such as smoking or exercise, were recorded. BMI, a statistical measurement that compares a person's weight and height, was calculated as weight in kilograms divided by the height in meters squared. Neck circumference was measured at the cricothyroid level. Sleepiness was evaluated by ESS. To exclude any respiratory or cardiovascular disease, spirometric evaluation, arterial blood gas analysis while the patient breathed room air, chest radiograph, and resting 12-lead electrocardiogram were conducted. All data were extracted from the medical records using a specially designed case report form (CRF). Then the data were double-entered manually into a Microsoft Excel master sheet to build our database. All forms were checked by another researcher for errors. The variables recorded on the CRF and pertinent definitions were described as follows.

#### 2.2.1. Sleep Study

Subjects underwent an overnight polysomnography (PSG) with the Embla-Monet 32 Sleep System (Embla, USA) which consisted of recording for EEG, electrooculography, electromyography, ECG, nasal pressure transducer, thermistor for nasal airflow, thoracic and abdominal impedance belts, pulse oximetry, tracheal microphone for snoring, and sensors for leg and sleep position. PSG recordings were manually scored by a single registered technologist as described previously [19–21].

Apnea was defined as the pause of airflow at the nose and mouth lasting for >10 s. Hypopnea was defined as a decrease of ≥30% in thoracoabdominal motion associated with a fall in baseline oxygen saturation of >4%. The apnea-hypopnea index (AHI) was expressed as the number of episodes of apnea and hypopnea per hour of total sleep time (TST). The severity of hypoxia was assessed by calculating the lowest oxygen saturation (nadir SaO_2_) and the percentage of TST spent with oxygen saturation at SaO_2_ < 90%. OSAS was defined by AHI ≥ 5/h. OSAS subjects were classified into two groups according to their apnea-hypopnea frequency as mild to moderate OSAS (AHI > 5 ⩽ 30), and severe OSAS (AHI > 30).

#### 2.2.2. Blood Samples and PBMC Isolation

Blood samples were obtained the morning after polysomnography, between 08:00 and 09:00 following an overnight fast. The 15–20 mL of peripheral blood (PB) samples were collected into collection tubes containing 0.2 mL sodium heparin. PBMCs were prepared by Ficoll-Hypaque density centrifugation for analysis of flow cytometry and real-time quantitative polymerase chain reaction (RT-qPCR). Plasma was obtained after centrifugation and stored at −80°C for the measurement of the cytokines.

#### 2.2.3. Flow Cytometric Analysis of Treg and Th17 Cells

PBMCs were suspended at a density of 2 × 10^6^ cells/mL in complete culture medium (RPMI 1640 supplemented with 100 U/mL penicillin, 100 *μ*g/mL streptomycin, 2 mM glutamine, 10% heat inactivated fetal calf serum; Gibco BRL, Gaithersburg, MD). The cell suspension was transferred to each well of 12-well plates. Cultures were stimulated with phorbol myristate acetate (PMA, 50 ng/mL) plus ionomycin (1 *μ*M) for 4 h in the presence of monensin (500 ng/mL; all from Alexis Biochemicals, San Diego, CA). The incubator was set at 37°C under a 5% CO_2_ environment. After 4 h of culture, the contents of the well were transferred to 5-mL sterile tubes and centrifuged at 1500 rpm for 5 min.

For the Th17 analysis, the cells were incubated with phycoerythrin (PE) antihuman CD4 (eBioscience, San Diego, CA) at 4°C for 20 min. For the Treg analysis, the cells were incubated with fluorescein isothiocyanate (FITC) anti-human CD4 and PE anti-human CD25 at 4°C for 30 min. Following surface staining, the cells were fixed and permeabilized according to the manufacturer's instructions, and then stained with FITC anti-human IL-17A for Th17 detection or PerCP-Cy5.5 (PC61.5; eBioscience) anti-human Foxp3 for Treg detection. Isotype controls were treated to enable correct compensation and confirm antibody specificity. All of the antibodies and reagents were from BD Pharmingen. Stained cells were analysed by flow cytometric analysis using a FACScan flow cytometer (Becton Dickinson) equipped with CELLQest Pro 5.2 software (BD Biosciences, USA).

#### 2.2.4. ROR*γ*t and Foxp3 Expression Determined by Real-Time Quantitative PCR

Total RNA was extracted from the PBMCs with TRIzol extraction (Invitrogen, Carlsbad, CA, USA) according to the manufacturer's instructions, cDNA was synthesized using random hexamer primers and RNase H-reverse transcriptase (Invitrogen). The following primer pairs were used: Foxp3: F: 5′-CACGCATGTTTGCCTTCTTCAGA-3′, R: 5′-GTAGGGTTGGAACACCTG CTGGG-3′, and ROR*γ*t: F: 5′-GCAATGGAAGTGGTGCTGGTT-3′, R: 5′-AGGATGCTTTGGCGATGAGT C-3′. Primers were entered into NCBI BLAST database to ensure that it was specific for the target mRNA transcription and then synthesized by Sangon Biotech Co. (Japan). PCR were performed using Syber Green real-time PCR regent box (Toyobo) in a total volume of 20 *μ*L. PCR conditions for Foxp3 was 95°C for 1 min, 40 cycles of 95°C for 15 s, 61°C for 15 s, 72°C for 45 s, but the annealing temperature of ROR*γ*t was 64°C. Samples were analyzed utilizing the ABI Prism 7900 Sequence Detection System (Applied Biosystems, Foster City, CA, USA). Relative gene expression was calculated by using the comparative CT method. Glyceraldehyde-3-phosphate dehydrogenase (GAPDH) was used as a housekeeping gene for normalization, and a no template sample was used as a negative control. All reactions were carried out in triplicate per sample.

#### 2.2.5. Detection of Plasma Cytokines and hsCRP

The plasma levels of IL-17, IL-6, TGF-*β*_1_, and IL-10 were measured by enzyme-linked immunosorbent assay (ELISA), following the manufacture's instructions (ELISA kits, all from R&D system). The minimal detectable concentrations were 2 pg/mL for IL-17, 0.7 pg/mL for IL-6, 5 pg/mL for TGF-*β*_1_ and 7.8 pg/mL for IL-10. All samples were measured in duplicate.

As previously described [21], serum levels of highly sensitive CRP (hsCRP) were measured with a particle-enhanced Immunoturbidimetry (Beijing O&D Biotech Company Ltd, Cox Bio China, Beijing, China). The lower limit of detection for hsCRP was 0.06 mg/L.

#### 2.2.6. Statistical Analysis

Values are expressed as mean ± standard deviation (SD) in the tables and figures. Differences between the values were determined using Student's *t*-test. Grouped data were analysed using a one-way analysis of variance (ANOVA) followed by the Student-Newman-Keuls test. When the equal variance test failed, a Mann-Whitney rank sum test was used. Spearman's correlation was used as a test of correlation between two continuous variables. A *P* value of <0.05 indicates significance. Statistical analysis was performed using a commercial software package (SPSS, version 15; SPSS; Chicago, IL).

## 3. Results

### 3.1. Basic Characteristics of Studied Subjects

A total of forty-four men with newly diagnosed OSAS were recruited. Five patients were diagnosed as mild OSAS and 18 patients were moderate OSAS. Thus, a total of twenty-three patients were enrolled as mild to moderate OSAS group and 21 patients were screened as severe OSAS group. Twenty health men were enrolled as the control group at the same time. Overall clinical characteristics are summarized in Table 1.

The mean AHI in OSAS group was 40.41 ± 20.68 and mean age of OSAS patients was 46.43 ± 18.22 years. As shown, EES score and PSG parameters were significantly higher in the OSAS group comparing with the control group (*P* < 0.05). However, no statistically significant differences were found between three groups from the standpoint of age and BMI (*P* > 0.05).

### 3.2. Th17 Frequency Was Increased in the Peripheral Blood of OSAS Patients

**Figure 1:**
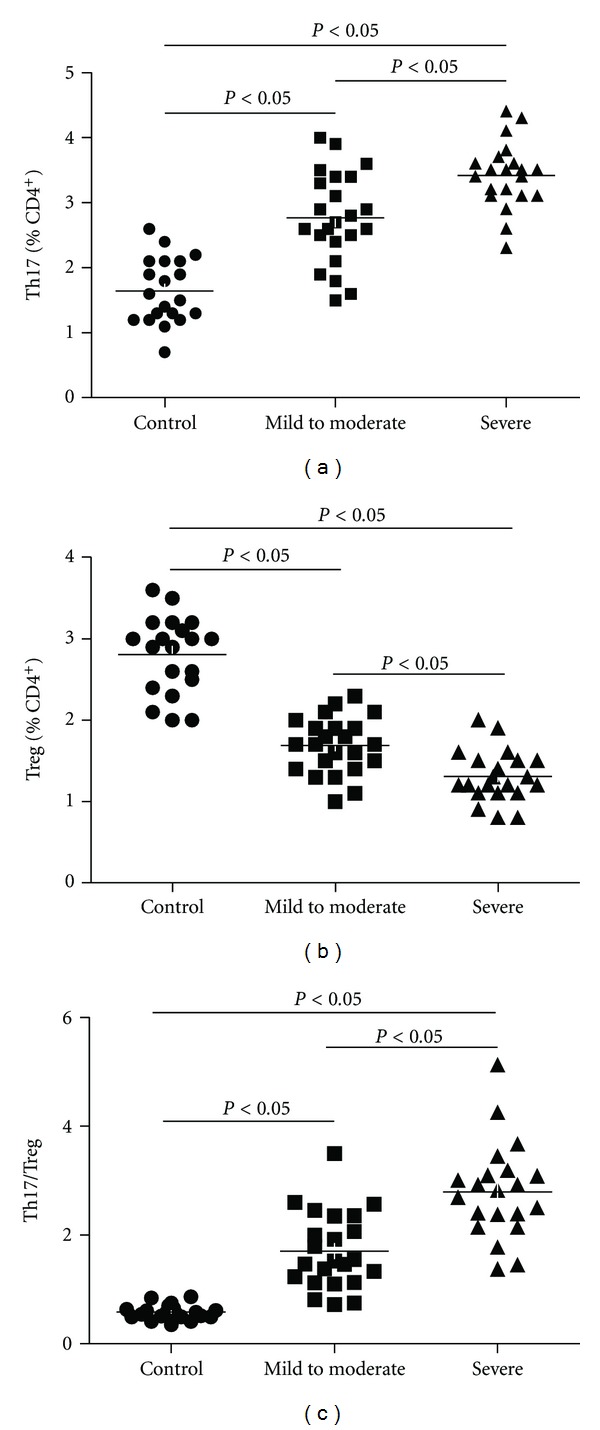
Frequencies of circulating Th17 and Treg cells, as well as the ratio of Th17/Treg in OSAS patients and control group. PBMCs from studied subjects were stained with labeled antibodies as described in Section 2. (a) Circulating Th17 frequencies increased in OSAS patients compared with controls; (b) circulating Treg cell subset decreased in OSAS patients compared with controls; (c) the ratio of Th17/Treg increased in OSAS patients compared with controls.

As shown in Figure 1(a), the prevalence of Th17 cells in PBMCs was expressed as a ratio of CD4^+^IL17^+^T cells/CD4^+^T cells. The frequence of Th17 was evidently increased in the peripheral blood of OSAS patients (3.08 ± 0.68%) than those in normal controls (1.65 ± 0.49%; *P* < 0.001). Furthermore, the percentage of Th17 was markedly higher in patients with severe OSAS (3.42 ± 0.49%) than those in subgroup with mild to moderate OSAS (2.77 ± 0.68%; *P* = 0.002).

### 3.3. Treg Frequency Was Decreased in the Peripheral Blood of OSAS Patients

The prevalence of Treg cells was expressed as a ratio of CD4^+^CD25^+^Foxp3^+^/CD4^+^T cells. As shown in Table 2 and Figure 1(b), the frequency of Treg was significantly decreased in the peripheral blood of OSAS patients (1.50 ± 0.38%) compared to control group (2.81 ± 0.46%; *P* < 0.001). Moreover, significant difference was found between severe OSAS group and patients with mild to moderate OSAS (1.30 ± 0.31% versus 1.69 ± 0.34%, *P* = 0.001). Nevertheless, the percentage of CD4^+^CD25^+^T lymphocytes was similar in the three groups (data not shown).

### 3.4. Balance of Th17 and Treg Was Disrupted in OSAS Patients

When the balance of Th17 and Treg was further investigated, their relationship was expressed as a ratio of Th17/Treg. We demonstrated that the ratio of Th17/Treg was highest in patients with severe OSAS (2.80 ± 0.84), lower in those patients with mild to moderate OSAS (1.71 ± 0.67) and lowest in control subjects (0.58 ± 0.13) (Figure 1(c)). As a result, the Th17/Treg ratio was significantly increased in OSA patients comparing with control group (2.23 ± 0.94 versus 0.58 ± 0.13, *P* < 0.001).

### 3.5. Expression of Foxp3 and ROR*γ*t mRNA in PBMCs

**Figure 2:**
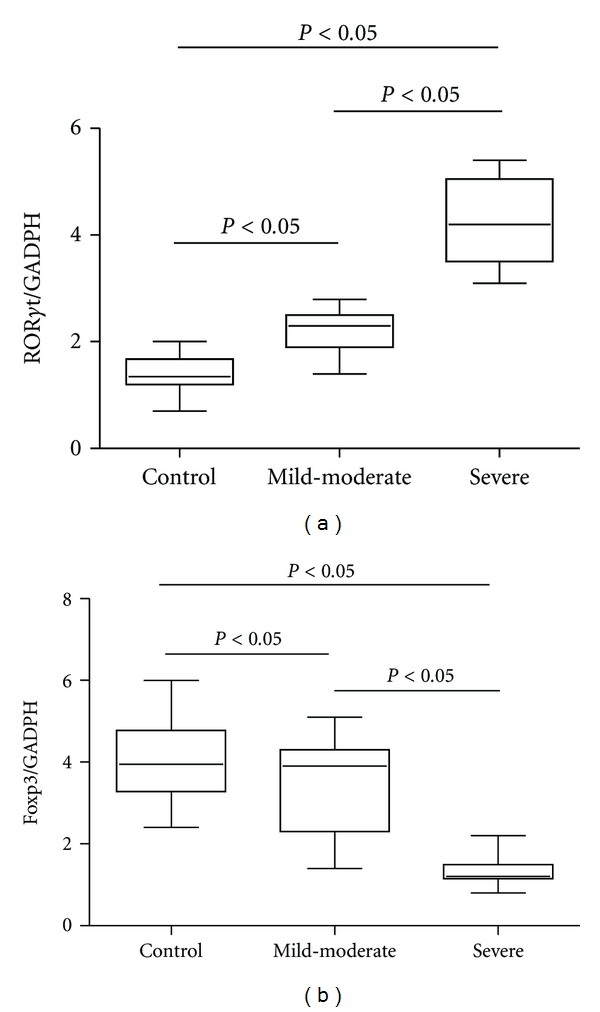
Expression of ROR*γ*t and Foxp3 in PBMCs from patients with OSAS and control groups. (a) Ratios of ROR*γ*t/GAPDH mRNA were compared in three groups. (b) Ratios of Foxp3/GAPDH mRNA were compared in three groups.

ROR*γ*t is an important transcription factor for the differentiation of Th17, while Foxp3 is the master transcription factor in Treg. We thus analyzed the levels of transcription factors for Th17 (ROR*γ*t) and Treg (Foxp3) by RT-qPCR in PBMCs from included subjects. As shown in Figure 2, the levels of ROR*γ*t expression were much upregulated in the OSAS group (3.18 ± 1.19) than those in the health control group (1.42 ± 0.32, *P* < 0.01). And there was significant difference between the severe (4.25 ± 0.79) and mild to moderate subgroups (2.21 ± 0.37) within OSAS patients (*P* < 0.05). In contrast, the expression of Foxp3 was markedly lower in the OSAS patients (2.50 ± 1.38) as compared with the control group (4.10 ± 0.97, *P* < 0.01), while Foxp3 levels in severe OSAS group (1.33 ± 0.35) were significantly downregulated than that of the subgroup with mild to moderate OSAS (3.56 ± 1.07, *P* < 0.01).

### 3.6. Plasma Concentrations of Related Cytokines and hsCRP

**Figure 3:**
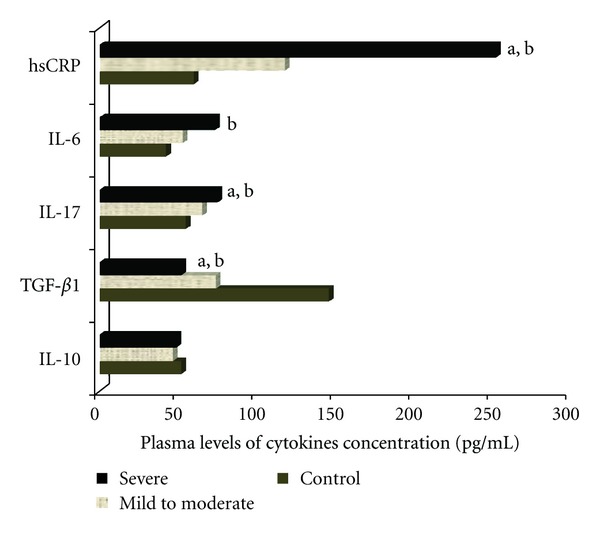
Represents the plasma levels of cytokines concentration in three groups (mild to moderate OSAS, severe OSAS and control group). The IL-17 and IL-6 concentrations in patients with OSAS were significantly higher when compared to concentrations in control group. While plasma TGF-*β*_1_ concentrations in the OSAS group were significantly lower than those of control group. And plasma IL-10 concentrations were comparable in three groups. ^a^ *P* < 0.05, Mild to Moderate OSAS versus Severe OSAS; ^b^ *P* < 0.05, Control versus Severe OSAS.

Plasma levels of IL-17, IL-6, TGF-*β*_1_, IL-10, and hsCRP were detected in normal controls and OSAS patients by means of ELISA tests (Table 2, Figure 3). IL-17 in severe patients (75.24 ± 11.40 pg/mL) and mild to moderate apnea (65.78 ± 13.97 pg/mL) were both significantly higher compared with concentration in control group (55.12 ± 18.23 pg/mL, *P* < 0.01). The level of IL-6 in OSAS patients (55.09 ± 17.02 pg/mL) was significant higher that in control group (42.56 ± 21.15 pg/mL, *P* < 0.01), however it's comparable in severe subgroup and patients with mild to moderate OSAS (*P* > 0.05). As shown in Figure 3, while plasma TGF-*β*_1_ in severe patients (52.19 ± 12.53 pg/mL) and mild to moderate patients (74.26 ± 11.40 pg/mL) with OSAS were markedly lower than those in the control group (146.81 ± 21.36 pg/mL; *P* < 0.01). In contrast, the same differences were not found for IL-10 (*P* > 0.05).

The levels of hsCRP were increased in OSAS group (183.11 ± 73.02 pg/mL) compared with those in control group (60.33 ± 20.38 pg/mL; *P* < 0.01). Moreover, hsCRP was markedly higher in subjects with severe OSAS (253.71 ± 36.49 pg/mL) when compared to subjects with mild to moderate OSAS (118.65 ± 16.72 pg/mL; *P* < 0.01).

### 3.7. Correlations between Peripheral Th17 Frequency and ROR*γ*t mRNA, Plasma IL-17 Level

**Figure 4:**
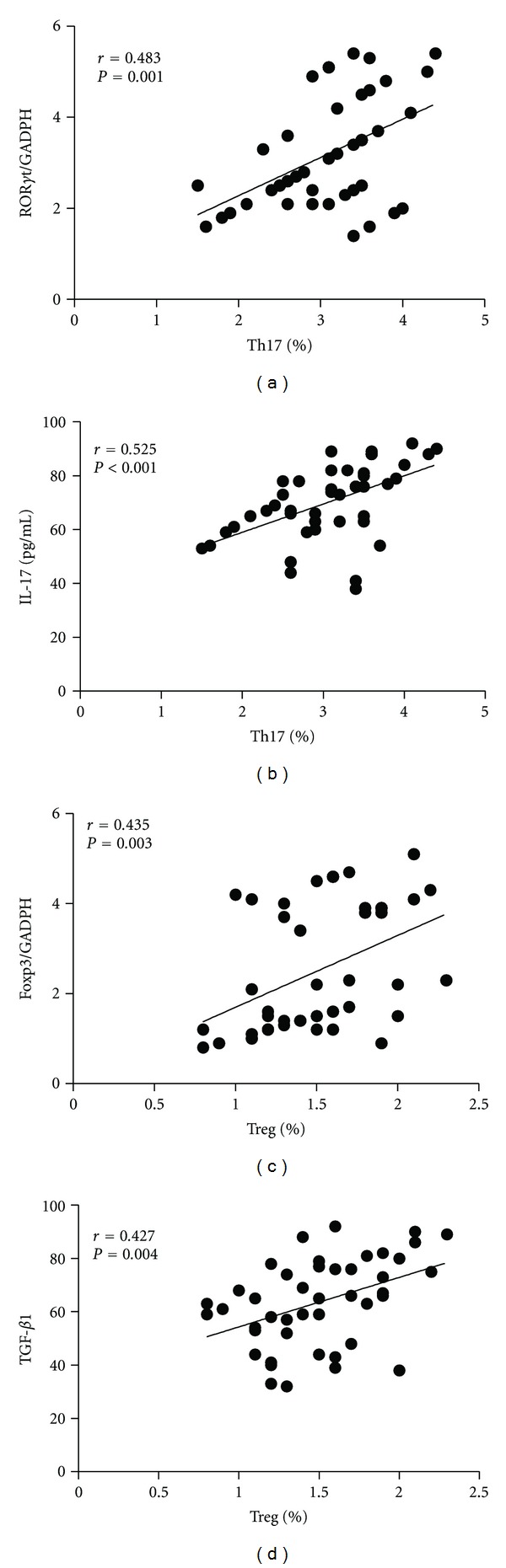
Spearman correlation of circulating cytokines and the frequences of Treg or Th17 cells in OSAS patients. (a) Ratios of ROR*γ*t/GAPDH mRNA positively correlate with Th17 percentage. (b) IL-17 concentrations positively correlate with Th17 percentage. (c) Ratios of Foxp3/GAPDH mRNA positively correlate with a proportion of Treg cells. (d) TGF-*β*_1_ concentrations positively correlate with a proportion of Treg cell.

In 44 patients with OSAS, the plasma concentration of IL-17 was positively correlated with peripheral blood frequencies of Th17 (*r* = 0.525, *P* = 0.000) (Figure 4(a)). There were significantly positive correlations between Th17 frequencies and the level of ROR*γ*t mRNA in PBMC from OSA patients (*r* = 0.483, *P* = 0.001) (Table 3, Figure 4(b)).

### 3.8. Correlations between Peripheral Treg Frequency and Foxp3 mRNA, Plasma TGF-*β*_1_ Level

And TGF-*β*_1_ concentration was positively correlated with peripheral blood frequencies of Treg (*r* = 0.427, *P* = 0.004) in OSAS patients (Figure 4(d)). Significantly positive correlations were found between peripheral Treg percentage and the expression of Foxp3 mRNA in PMBCs from OSAS patients (*r* = 0.435, *P* = 0.003) (Figure 4(c)). The correlations between other concentrations were all negative.

### 3.9. Correlation of Circulating Th17/Treg Ratios with hsCRP and Disease Severity in OSAS Patients

We next sought to analyze the correction of the ratio of Treg to Th17 cells to the disease severity in OSAS patients (Table 3). The plasma level of hsCRP has been proved to have a close relationship with severity and complication in OSAS patients. In current OSAS patients, serum level of hsCRP was positively correlated with AHI (*r* = 0.458, *P* < 0.001). In contrast, serum level of hsCRP was positively related with Th17 frequence (*r* = 0.372, *P* = 0.013) and negatively related with Treg frequence (*r* = −0.433, *P* = 0.003). And also, hsCRP level was positively correlated with the ratio of Th17/Treg (*r* = 0.475, *P* = 0.001) in OSAS patients.

As shown in Table 3, AHI index was positively correlated with circulating Th17/Treg ratio (*r* = 0.321, *P* = 0.029), but not with single percentage of Th17 or Treg in PBMC from OSAS patients (*P* > 0.05). This result suggests that decreased ratio of Treg to Th17 cells may be an indicator for the disease severity of OSAS indicated by AHI. There was no significant correlation between the other PSG parameters and the ratio of Th17 to Treg in the OSAS patients (*P* > 0.05). The relationship between BMI or ESS scores and Th17/Treg ratios in OSAS patients was also studied, but no correlations were identified (*P* > 0.05).

## 4. Discussion

The present data provide direct evidence that OSAS patients exhibited a significant increase in peripheral Th17 frequency, Th17-related cytokines (IL-17) and transcription factor (ROR*γ*t) levels, and dramatic decrease in Treg frequency, Treg-related cytokines (TGF-*β*_1_) and transcription factor (Foxp3) levels when compared to the control group. It's the first clue that circulating Treg/Th17 balance is impaired in these patients. More important, circulating Treg/Th17 ratio negatively correlated with the plasmatic hsCRP level and severity of OSAS, as determined by AHI. These results suggest a potential role for a higher ratio of circulating Th17/Treg, which indicates the systemic inflammation in the pathogenesis of OSAS and subsequent complications.

To the best of our knowledge, this is the first study to examine the circulating Th17 and Treg cells and their balance in OSAS patients. Bollinger et al. demonstrated that CD4^+^CD25^+^ natural regulatory T cells (nTreg) number and function follow a rhythm across the 24-h period and sleep deprivation severely disturbs the functional rhythm of nTreg cells [8]. In agreement, we found that the frequencies of Treg cells and the expression of Foxp3 were significantly lower in patients with OSAS when compared to normal persons. By far, there is no research investigating the role of peripheral frequence of Th17 cells in any sleep disorders. In this study, we found that patients with OSAS exhibited a significant increase in peripheral Th17 number, Th17-related cytokines (IL-17) and ROR*γ*t levels when compared to normal persons. As a result, a Treg/Th17 functional imbalance exists in OSAS patients, suggesting a potential role for Treg/Th17 imbalance in the pathogenesis of OSAS.

Th17 cells and Treg cells not only share the same origin but also are mutually antagonistic in function. Th17 cells have been established as an important T-helper effectors lineage, which mediates protection against extracellular pathogens. On the negative side, Th17 cells also appear to be the driving force in the pathogenesis of autoimmune and inflammatory disorders. In contrast to Th17 cells, CD4^+^CD25^+^ and FOXP3^+^ (Tregs) play a critical role in maintaining self-tolerance and in preventing organ-specific autoimmunity, allergy, and allograft rejection. T cell development exhibits a degree of plasticity that meets local requirements and thereby transgresses lineage barriers. Although these two T cell subsets have a reciprocal relationship and differentiation pathways, it was recently reported that Tregs are able to differentiate into IL-17-producing cells, and that human peripheral blood and lymphoid tissue contain a significant number of CD4^+^Foxp3^+^ T cells that have the capacity to produce IL-17 upon activation [22].

Especially, for the first time, we report that the ratio of Th17/Treg cells, but not peripheral Th17 or Treg frequence alone, had a linear negative correlation with OSAS severity indicated by AHI. In accordance to our results, Freire et al. found no association of AHI with total lymphocyte, neutrophil, or peripheral blood cell count alterations [9]. Recently, many investigators raised the notion of a Th17/Treg balance and reported an imbalance in patients with various autoimmune and inflammatory diseases [14–17]. A higher Th17/Treg ratio may characterize a more severity in autoimmune, inflammatory and allergic diseases. It illustrates that the balance or interplay between various types of immune cells may be the better predictors for clinical outcome of patients [23]. Our data may be compatible with this new theory. The ratio of Treg/Th17 may reflect a specific skewed balance of anti- and proinflammatory T cell subsets in OSAS patients, while absolute numbers reflect the general activation. Because of the reciprocal developmental pathway for the generation and the opposite effects of Th17 and Treg cells, Treg/Th17 subsets may therefore have been evolved to regulate systematic inflammation, analogous to the dichotomy of Th1/Th2 T-cell subsets. As a result, the Th17/Treg ratio became an important tool in describing and understanding various immunological conditions, such as tumor, inflammatory, autoimmune, and allergic disease or models.

Adenoid hypertrophy is the most common cause of upper airway obstruction and sleep-disordered breathing in children. Sade et al. evaluated the adenoidal lymphocyte subsets to describe the percentage of various lymphocyte subsets in hypertrophied adenoids of children and correlated them with symptom severity. They found a significant negative linear correlation between the Th17/Treg ratio and the patients' clinical scores independent of age and gender [24]. Apparently, our observation was opposite to the results of Sade. However, the pathogenesis and pathophysiology of OSAS in adult and children were not completely identical. Moreover, it may reflect the inconsistence between local and systemic inflammation in OSAS. In the context of acute and chronic infectious existing in local adenoids of children, a lower Th17/Treg ratio might decrease the total clearance of microorganisms and increase chronic immune activation and proliferation of lymphocytes, thereby causing hypertrophy of lymph nodes, including adenoids and tonsils. It has recently been hypothesized that adult OSAS might be a triggering factor for the development of autoimmune phenomena [25]. Under the circumstances, a higher Th17/Treg ratio may bring on the loss of tolerance and regulation, and finally a persistent, low-degree systemic inflammatory reaction characterized by autoimmune or allergic diseases.

Activation of T lymphocytes is among the crucial steps leading to the release of inflammatory mediators and adhesion molecules. Activation of inflammatory cells and their interaction with endothelial cells have been demonstrated in OSAS [26]. In a series of experiments, Dyugovskaya et al. showed that various subpopulations of cytotoxic T cells of patients with OSAS acquire an activated phenotype with the downstream consequence of increased cytotoxicity against endothelial cells [10, 11]. The research in the past five years have demonstrated that subsets of T lymphocytes, both effector T cells such as Th1 and Th2 lymphocytes, as well as Th17 and Treg, play critical roles in the development of atherogenesis and vascular remodeling [27, 28].

Several large-scale prospective studies have shown that the hsCRP level is an independent predictor of risk of myocardial infarction, stroke, peripheral vascular disease, and vascular death, making it a useful marker for cardiovascular risk stratification [29]. Previous works have demonstrated an independent relationship between OSAS and an elevated hsCRP [30, 31]. A close link between circulating Treg/Th17 ratio and serum hsCRP level had been proved in our paper. Kim et al. recently reported that FOXP3 DNA methylation levels positively correlated with hsCRP levels and AHI in 47 OSAS children [32]. A high FOXP3 DNA methylation may favor the downregulation of Foxp3 protein expression and thus reduce the number of Tregs, thereby favoring increased systemic inflammatory responses in pediatric OSAS. Accordingly, their results corroborated our observation that the ratio of Treg/Th17 was negatively correlated with hsCRP and AHI in OSAS patients. It should be careful in understanding and comparing previous results because only sensitive measurement of hsCRP was valid to demonstrate an association with AHI.

The cytokine milieu of the local environment plays a pivotal role in the differentiation from naive CD4^+^ T cells to Treg or Th17 cells. In the present study, results showed that the concentrations of IL-6 and IL-17, both of which promote the differentiation of Th17 cells, were all significantly higher in OSAS patients, and IL-17 concentrations were positively correlated with Th17 cells frequencies. These suggested that the proinflammatory cytokine microenvironment, characterized by elevated IL-6 and IL-17 levels and decreased TGF-*β*_1_ concentrations, could potentially support the continued generation of Th17 cells but meanwhile suppress the development of Treg cells, which led to the Th17/Treg numerical imbalance in OSAS patients [33, 34]. This numerical imbalance might consequently result in the functional imbalance of Th17/Treg which contributed to enhancing the formation of the inflammatory cytokine microenvironment, and eventually formed a positive feedback mechanism to amplify proinflammatory immune responses.

A potential reason for the immune system activation in OSAS patients may be the obesity that represents a major risk factor and usually accompanies OSAS [35]. Although the BMI levels remained comparable, there were clearly some overweight men with a mean BMI around 28 kg/m^2^ were included in our cohort. Further research concerning this issue may enroll only non-overweight OSAS groups for analysis. And other measurements, such as hip-waist ratio or densitometry of abdominal fat, can replace BMI to assess obesity in OSAS patients.

## 5. Conclusion

Our data offered direct evidence for the skewed balance of Th17/Treg, pro- and anti-inflammatory T cell subsets in OSAS patients. We also identified that the ratio of Th17/Treg does not only correlate with inflammatory marker but is also associated with disease severity of OSAS. Thus, our results suggest that increased ratios of Th17/Treg subpopulations may play a role in the pathogenesis of OSAS. These results opened an alternative explanation for the substantial activation of immune cells as well as the development of OSAS and complications, which may have significant impacts on the prevention and treatment of OSAS patients.

The biological significance and clinical implications of the imbalance of serum Th17/Treg merit future confirmatory and mechanistic studies in larger cohorts. The further studies may be designed to observe the change of Treg/Th17 imbalance in OSAS during constant positive airway pressure (CPAP) treatment and to investigate the impact of comorbidies on the Treg/Th17 balance in OSAS patients. Restoration of the immune imbalance may be a future therapeutic approach for inhibiting systemic inflammatory processes in order to ameliorate the cardiovascular risk factors associated with OSAS.

## Figures and Tables

**Table 1:** Baseline characteristics of study population.

Variables	Control	OSAS		
		Total	Mild to Moderate	Severe
Subjects, *n *	20	44	23	21
Age (years)	45.80 ± 23.01	46.43 ± 18.22	44.90 ± 20.64	48.07 ± 11.28
BMI (kg/m^2^)	26.90 ± 4.25	28.15 ± 5.34	28.55 ± 6.29	27.82 ± 5.01
Neck circumference (cm)	41.15 ± 5.63	43.93 ± 5.72	43.58 ± 6.22	44.13 ± 5.19
AHI (episodes/h)	2.26 ± 1.30	40.41 ± 20.68*	23.12 ± 6.93^‡^	59.33 ± 12.59^†§^
Nadir SaO_2_ (%)	94.78 ± 3.49	76.24 ± 7.56*	80.65 ± 8.19^‡^	71.56 ± 7.87^†§^
SaO_2_ < 90% (%TST)	1.06 ± 0.93	17.63 ± 8.32*	7.72 ± 7.43^‡^	28.52 ± 10.37^†§^
ESS scores	4.93 ± 2.05	14.74 ± 1.74*	13.80 ± 1.92^‡^	15.95 ± 1.63^§^

Values are expressed as mean ± SD.

*P < 0.05, Control versus total OSAS;

^†^ *P* < 0.05, Mild to Moderate OSAS versus Severe OSAS;

^‡^ *P* < 0.05, Control versus Mild to Moderate OSAS;

^§^ *P* < 0.05, Control versus Severe OSAS.

Definition of abbreviations: AHI: apnoea hypopnoea index; BMI: body mass index; ESS, Epworth sleepiness scale; nadir: lowest oxygen saturation recorded; OSAS: obstructive sleep apnoea syndrome; TST: total sleep time.

**Table 2:** Summary of RT-qPCR, flow cytometry, and ELISA results in study population.

Variables	Control	OSAS (*n* = 44)		
		Total	Mild to Moderate	Severe
Subjects, *n *	20	44	23	21
T-cell counts (Flow cytometry)				
Th17 (% of CD4^+^)	1.65 ± 0.49	3.08 ± 0.68*	2.77 ± 0.68^‡^	3.42 ± 0.49^†§^
Treg (% of CD4^+^)	2.81 ± 0.46	1.50 ± 0.38*	1.69 ± 0.34^‡^	1.30 ± 0.31^§^
Th17/Treg ratio	0.58 ± 0.13	2.23 ± 0.94*	1.71 ± 0.67^‡^	2.80 ± 0.84^†§^
mRNA expression of transcription factors (RT-qPCR)				
ROR*γ*t /GADPH mRNA	1.42 ± 0.32	3.18 ± 0.19*	2.21 ± 0.37^‡^	4.25 ± 0.79^†§^
Foxp3/GADPH mRNA	4.10 ± 0.97	2.50 ± 1.38*	3.56 ± 1.07^‡^	1.33 ± 0.35^†§^
Protein levels of cytokines (pg/mL) (ELISA)				
IL-10	52.17 ± 23.18	47.84 ± 10.72	46.79 ± 11.69	49.06 ± 8.51
TGF-*β*_1_	146.81 ± 21.36	63.18 ± 16.10*	74.26 ± 11.40^‡^	52.19 ± 12.53^†§^
IL-17	55.12 ± 18.23	70.30 ± 13.65*	65.78 ± 13.97^‡^	75.24 ± 11.40^†§^
IL-6	42.56 ± 21.15	55.09 ± 17.02*	56.20 ± 22.41^‡^	53.75 ± 19.52^§^
hsCRP	60.33 ± 20.38	183.11 ± 73.02*	118.65 ± 16.72^‡^	253.71 ± 36.49^†§^

Values are expressed as mean ± SD.

The prevalence of Treg or Th17 cells was expressed by a percentage of CD4^+^CD25^+^Foxp3^+^/CD4^+^T or CD4^+^IL-17^+^/CD4^+^ T lymphocytes.

*P < 0.05, Control versus total OSAS;

^†^ *P* < 0.05, Mild to Moderate OSAS versus Severe OSAS;

^‡^ *P* < 0.05, Control versus Mild to Moderate OSAS;

^§^ *P* < 0.05, Control versus Severe OSAS.

**Table 3:** Correlation coefficients between circulating Th17, Treg, or ratio and BMI, PSG parameters, and inflammatory cytokines in OSAS patients (*n* = 44).

Parameters	Th17		Treg		Th17/Treg	
	*r *	*P *	*r *	*P *	*r *	*P *
BMI	0.124	NS	0.140	NS	0.260	NS
AHI	0.284	NS	−0.290	NS	0.321	0.029
Nadir SaO_2_ (%)	0.189	NS	−0.238	NS	0.194	NS
SaO_2_ < 90% (%TST)	0.207	NS	−0.274	NS	0.262	NS
ESS scores	0.175	NS	−0.266	NS	0.178	NS
ROR*γ*t/GADPH mRNA	0.483	0.001	−0.114	NS	0.076	NS
Foxp3/GADPH mRNA	−0.234	NS	0.435	0.003	−0.053	NS
IL-10 (pg/mL)	0.089	NS	0.120	NS	0.095	NS
TGF-*β*_1_ (pg/mL)	−0.291	NS	0.427	0.004	−0.211	NS
IL-17 (pg/mL)	0.525	<0.001	−0.224	NS	0.288	NS
IL-6 (pg/mL)	0.068	NS	0.106	NS	0.183	NS
hsCRP (pg/mL)	0.372	0.013	−0.433	0.003	0.475	0.001

Definition of abbreviations:

AHI: apnoea hypopnoea index; BMI: body mass index; hsCRP: high-sensitivity C-reactive protein; ESS: Epworth sleepiness scale; nadir: lowest oxygen saturation recorded; OSAS: obstructive sleep apnoea syndrome; TST: total sleep time.

## Abbreviations

AHI: Apnoea-hypopnoea index
BMI: Body mass index
CPAP: Continuous positive airway pressure
CRF: Case report form
ESS: Epworth Sleepiness Scale
Foxp3: Forkhead box P3
hsCRP: High-sensitivity C-reactive protein
OSAS: Obstructive sleep apnoea syndrome
PBMC: Peripheral blood monouclear cells
PSG: Polysomnography
ROR*γ*t: Retinoic acid related orphan receptor *γ*t
TST: Total sleep time.
